# Comparative Analysis of γ-Oryzanol, β-Glucan, Total Phenolic Content and Antioxidant Activity in Fermented Rice Bran of Different Varieties

**DOI:** 10.3390/nu9060571

**Published:** 2017-06-03

**Authors:** Tae-Dong Jung, Gi-Hae Shin, Jae-Min Kim, Sun-Il Choi, Jin-Ha Lee, Sang Jong Lee, Seon Ju Park, Koan Sik Woo, Sea Kwan Oh, Ok-Hawn Lee

**Affiliations:** 1 Department of Food Science and Biotechnology, Kangwon National University, Chuncheon 24341, Korea; lgtjtd@naver.com (T-D.J.); cordelia162@hanmail.net (G-H.S); akxlwmf@nate.com (J-M.K.); docgotack89@hanmail.net (S-I.C.); tre98@hanmail.net (J-H.L.); 2 STR Biotech Company, LTD., Chuncheon 24232, Korea; sj@strbiotech.co.kr (S.J.L.); coco4649@strbiotech.co.kr (S.J.P.); 3 Crop Post-harvest Technology Division, Department of Central Area Crop Science, National Institute of Crop Science, RDA, Suwon 16613, Korea; wooks@korea.kr (K.S.W.); ohskwan@korea.kr (S.K.O.)

**Keywords:** rice bran, fermented, cultivars, antioxidant activity, γ-oryzanol

## Abstract

Rice bran, a by-product derived from processing rice, is a rich source of bioactive compounds. Recent studies have suggested that the fermentation can improve their biological activities. This study aimed to determined the level of γ-oryzanol, β-glucan and total phenol contents of fermented rice bran from 21 Korean varieties, as well as to evaluate their antioxidant activities. We also assessed the validation of the analytical method for determining γ-oryzanol content in fermented rice brans. Among the fermented rice brans, the Haedam rice bran contained the highest level of total phenol content (156.08 mg gallic acid equivalents/g), DPPH (2,2-diphenyl-1-picrylhydrazyl) radical scavenging activity (71.30%) and ORAC (Oxygen radical absorbance capacity) value (1101.31 μM trolox equivalents/g). Furthermore, the fermented Migwang rice bran showed the highest level of γ-oryzanol content (294.77 ± 6.74 mg/100 g).

## 1. Introduction

Rice (*Oryza sativa* L.) bran is a by-product produced in processing rice. Traditionally, rice bran, produced as a by-product during milling, is considered as waste. However, it is a fact that rice bran consists of up to 10% of rough rice and is a rich natural source of important antioxidant polyphenolics, flavonoids, phytic acid, vitamin E and γ-oryzanol [1]. γ-Oryzanol is a mixture of ferulic acid esters of triterpene alcohols [2]. The four major constituents are cycloartenyl ferulate, cyclobranol ferulate, campesterol ferulate and β-sitosterol ferulate [3]. γ-Oryzanol has been reported to have some biological activities; for example, improvement of plasma lipid pattern, inhibition of the platelet aggregation, reduction of total plasma cholesterol and increase of high density lipoprotein (HDL) cholesterol levels [4]. Rice bran was reported to contain a significant level of total phenolic content, DPPH (2,2-diphenyl-1-picrylhydrazyl) radical scavenging capacities and γ-oryzanol contents and these levels are significantly different among the varieties [5].

An antioxidant in the food science field is defined as any substance that is able to decrease or delay the oxidation of cellular oxidizable substances induced by reactive oxygen species (ROS) such as superoxide, hydrogen peroxide, singlet oxygen and hydroxyl radical [6]. Synthetic antioxidants including propyl gallate (PG), butylated hydroxyanisole (BHA), butylated hydroxytoluene (BHT) and tert-butylhydroquinone (TBHQ) are the most commonly used in food additives. However, many researchers have reported adverse effects of synthetic antioxidants such as toxic and carcinogenic effects [7]. Hence, the interest in natural and safer antioxidants for food applications has increased and there is a growing trend in consumer preferences for natural antioxidants. These trends have given an impetus to explore natural sources of antioxidants [8].

Bioconversion is a technique that may produce beneficial bioactive compounds during fermentation using microorganisms and enzymes. Two fermentation techniques, solid-state fermentation (SSF) and submerged liquid fermentation (SLF), have been used to culture mycelia. The operation of the scaled-up SSF system is complicated because of the difficulty in monitoring and controlling factors such as operating temperature and pH, whereas the SLF system provides good mixing conditions during bioprocessing [9]. Moreover, because the homogeneity enables valuable monitoring and efficient control of these factors, scaling up mycelial cultivation is easier for the SLF system than that of the SSF system [10]. Recently, many studies have shown that SSF of dietary fungus could induce enzyme activity which could also lead to the release of phenolic antioxidants [11]. In addition, being fermented by *Lentinula edodes* could enhance the antioxidants [12].

The aim of this study was to analyze the bioactive compounds (γ-oryzanol, β-glucan and total phenol contents) and antioxidant activities (DPPH radical scavenging and oxygen radical absorbance capacity (ORAC) value) of 21 varieties of fermented rice bran by bioconversion with *L. edodes* mushroom mycelia in the SLF system. Furthermore, to validate the HPLC method for γ-oryzanol content, we confirmed the linearity, the specificity, the limit of detection (LOD), the limit of quantification (LOQ), the precision and the accuracy.

## 2. Materials and Methods 

### 2.1. Chemicals 

Sodium carbonate, Folin Ciocalteu’s phenol reagent, gallic acid, DPPH, sodium phosphate dibasic, 6-hydrolxy-2,5,7,8,-tetramethylchroman-2-carboxylic acid (Trolox) and 2,2’-azobis (2-methylpropionamidine) dihydrochloride (AAPH) were purchased from Sigma-Aldrich Co. (Saint Louis, MO, USA). Potassium phosphate monobasic and fluorescein (sodium salt) were obtained from Junsei Chemical (Tokyo, Japan). The mixed-linkage beta-glucan assay kit was from Megazyme (The Bray Co., Wicklow, Ireland) and the γ-oryzanol standard was purchased from Wako Pure Chemical Industries, Ltd. (Tokyo, Japan) at a purity of 98.0%. HPLC-grade methanol, acetonitrile and isopropanol were obtained from J. T. Baker (Phillipsburg, NJ, USA).

### 2.2. Sample Preparation

Twenty one rice bran varieties (Table 1) were harvested during October, 2014 and supplied by the Korean Rural Developmental Administration (KRDA, Suwon, South Korea). Rice bran was fermented with *L. edodes* using the submerged liquid fermentation system by STR Biotech Ltd. (Chuncheon, South Korea). Briefly, *L. edodes* fungal mycelia were isolated from the mushroom fruit body and cultured on potato dextrose agar medium (PDA, Difco Laboratory, Detroit, MI, USA). The genetic identity of the fungus was confirmed by the Korean Center of Microorganisms (Seoul, South Korea). The mycelia cultured on PDA media were inoculated in 50 mL of the liquid media containing 2% of glucose, 0.5% of yeast extract, 0.5% of soy peptone, 0.2% of KH_2_PO_4_, 0.05% of MgSO_4_ and 0.002% of FeSO_4_. The culture experiments were conducted in 250 mL Erlenmeyer flasks at 28 °C for 5 days in a rotary shaker (120 rpm) to be used to seed the main liquid culture. The main liquid media containing rice bran (20 g/L) was treated with amylase and cellulose at 60 °C for 60 min for enzymatic digestion of particular materials containing carbohydrate. Subsequently, the culture mass was adjusted to pH 6.0 with HCl and sterilized in the autoclave. The experiment with the main liquid culture was started using a 5 L fermentor (working volume of 3 L) at 28 °C and 150 rpm by inoculating with the inoculum (10%) of the preliquid cultured mycelia. After 7 days, an enzyme mixture for lysis of cell wall containing cellulose, hemicellulose, pectinase, β-glucanase, mannose, and arabinose was kept at 50 °C for 60 min. Then, the enzyme-treated culture mass was extracted at 90 °C for 60 min and frozen and dried to a solid material. Each non-fermented or fermented rice bran (2.5 g) was mixed with 50 mL of methanol and sonicated for 60 min. The mixture was centrifuged at 3000× *g* for 10 min and the supernatant was filtered through 0.45 μm PVDF filter (Millex-HV, Millipore, Bedford, MA, USA).

### 2.3. Analysis of γ-Oryzanol Content

The γ-oryzanol content of rice bran was analyzed by reverse-phase HPLC system (Waters 2695 Separation Module, Waters Co., Milford, MA, USA) equipped with a binary pump, an auto sampler, a column heater and a PDA detector [13]. γ-Oryzanol was separated on a Sunfire C18 column (Waters, 4.6 mm × 250 mm, 5.0 μm) maintained at 40 °C using an isocratic mobile phase of acetonitrile/methanol/isopropanol (50:45:5 by volume) on a flow rate of 1.0 mL/min. The injection volume was 10 μL and the detection was completed on 320 nm. Acquisition and remote control of data from the HPLC system were performed using Empower software. The HPLC method for detection and quantification γ-oryzanol in rice bran was validated in terms of linearity, specificity, precision, accuracy, limit of detection (LOD) and limit of quantification (LOQ) according to the guidelines of the International Conference on Harmonization [14]. Calibration curves from each validation were evaluated to ensure that linearity and specificity were consistent with observations during the developing method. The specificity was evaluated with peak retention time and chromatogram detected γ-oryzanol was evaluated using the PDA detector recording the corresponding UV spectra at different points. In the assessment of linearity, calibration curve was plotted 7.8125, 15.625, 31.25, 62.5, 125, 250 μg/mL for γ-oryzanol. The precision (repeatability, expressed as RSD, %) and the accuracy were determined on spikes for γ-oryzanol at three different concentration levels (50, 60, and 75 μg/mL). This experiment was performed by intra-day (three repetitions on a single day) and inter-day (three repetitions on three different days). The LOD and LOQ were calculated for each analyte in all of commodities including non-fermented and fermented rice brans according to the following equations: LOD = 3.3 σ/S, LOQ = 10 σ/S, where σ is the mean standard deviation and S is the lope of the same equation, respectively.

### 2.4. Analysis of Total Phenol Content (TPC)

The total phenol contents were determined using Folin Ciocalteu’s colorimetric method [15]. One milliliter of the sample or standard (gallic acid) was mixed with 1 mL of 2% of sodium carbonate solution and 1 mL of 10% of Folin Ciocalteu’s phenol reagent. After 60 min, the absorbance was measured at 750 nm using a microplate reader (Molecular Devices, Sunnyvale, CA, USA). The measurement was compared to a calibration curve of gallic acid and the results were expressed as milli-grams of gallic acid equivalents (GAE) per gram of sample (mg GAE/g).

### 2.5. Analysis of β-Glucan Content

β-glucan was measured a mixed-linkage beta-glucan assay kit from Megazyme, which uses purified enzymes, according to the manufacturer’s protocol. For the β-glucan content, the sample (50 mg) was mixed with 0.1 mL of aqueous ethanol (50% v/v) and 2 mL of sodium phosphate buffer (20 mM, pH 6.5) in a boiling water bath, then incubated and stirred at 100 °C for 3 min. Then, the sample was incubated with lichenase (0.1 mL, 10 U) at 50 °C for 60 min and cooled down at room temperature for 5 min. After adding 2.5 mL of sodium acetate buffer (200 mM, pH 4.0), the prepared sample was centrifuged at 1500× *g* for 10 min. The supernatant (0.05 mL) was added into β-glucosidase (0.1 mL, 0.2 U) plus 0.05 mL of sodium acetate buffer (50 mM, pH 4.0) at 50 °C for 10 min. For quantification of glucose in the reaction solution, 1.5 mL of GOPOP (glucose oxidase/peroxidase) reagent was added and incubated at 50 °C for 20 min. The absorbance was measured at 510 nm using a spectrophotometer (Optizen POP, Mecasys Co., Ltd., Daejeon, South Korea). 

### 2.6. Determination of Antioxidant Activity

DPPH radical scavenging activity was determined according to the method [16] with some modifications. Two hundred micro liters of sample was added to 800 μL of 0.4 mM of DPPH solution and mixed thoroughly. The mixture was allowed to react at room temperature for 10 min, protected from light. The absorbance was then measured at 490 nm using a microplate reader. The results were calculated upon the following formula.

DPPH radical scavenging activity (%) = (1 − A_Experiment_/A_Control_) × 100

The ORAC assay was analyzed according to Zulueta et al. [17] with some modifications. Samples were diluted with 75 mM of potassium sodium phosphate buffer (pH 7.4). Twenty five microliter of diluted samples, Trolox (0–10 μM) or potassium sodium phosphate buffer (blank) and 150 μL of fluorescein (40 nM) were carried out in black 96-well plates. In addition, 25 μL of AAPH (18 mM) was pre-warmed at 37 °C for 15 min and then transferred to each well. The absorbance was immediately read by a fluorescence microplate reader. The analyzer is designated to register at 485 nm of an excitation wavelength and at 530 nm of an emission wavelength every 3 min for 90 min at 37 °C. The values of the ORAC assay were calculated by a calibration curve of Trolox and an area under the fluorescence decay curve. The ORAC values were expressed as Trolox equivalents as micro mole per gram (μM TE/g).

### 2.7. Statistical Analysis

All of the data were presented as means ± SD of triplicated samples. The data were analyzed using one-way ANOVA procedure of SAS 9.3 (SAS Institute Inc., Cary, NC, USA). Differences were analyzed by Duncan’s multiple range tests at *p* < 0.05. Correlations were calculated with Pearson’s correlation coefficient (*r*).

## 3. Results and Discussion

### 3.1. γ-Oryzanol Content of Non-Fermented and Fermented Rice Brans

**Figure 1:**
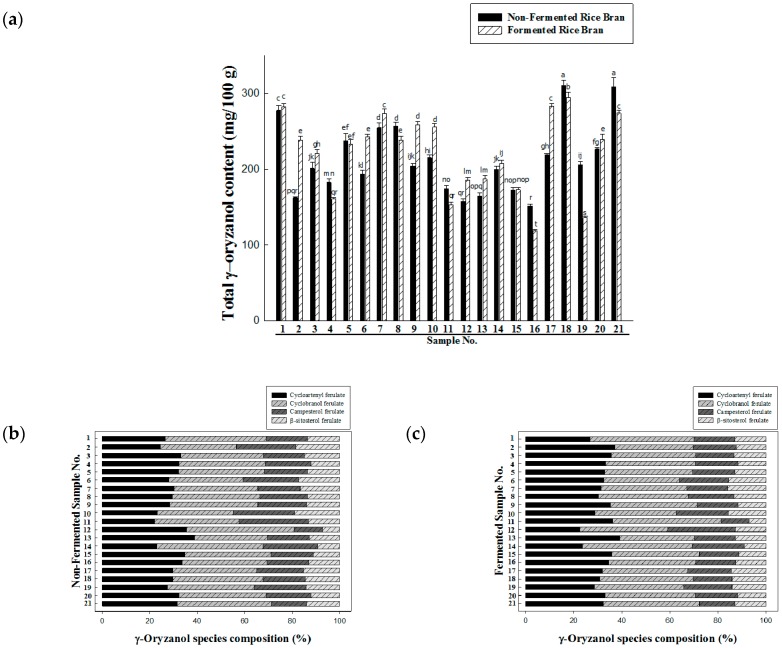
Total γ-oryzanol content. Comparison of non-fermented and fermented rice bran (**a**); composition of γ-oryzanol species of non-fermented rice bran (**b**); and fermented rice bran (**c**).

The change by bioconversion in γ-oryzanol content of 21 rice brans is shown in Figure 1a. γ-Oryzanol content was estimated by the standard curve (*y* = 24316*x* + 12935, *R*^2^ = 0.9999). The levels of γ-oryzanol of non-fermented and fermented rice brans ranged from 118.80 to 311.07 mg/100 g and the highest value was non-fermented Migwang rice bran (No. 18, 311.07 ± 7.07 mg/100 g) followed by fermented Haiami rice bran (No. 21, 309.25 ±12.20 mg/100 g) and fermented Migwang rice bran (No. 18, 294.7748 ± 6.74 mg/100 g). However, the changes of γ-oryzanol level by bioconversion in rice brans were not significant but significant differences on the proportion among four γ-oryzanol species in different cultivars in this study (Figure 1b,c), interestingly. For example, the proportions of cyclobranol ferulate in non-fermented and fermented Dasan1 rice bran (No. 14), fermented Anda rice bran (No. 11), non-fermented Danmi rice bran (No. 12), non-fermented and fermented Segyejinmi rice bran (No.1) were remarkably higher than those of other cultivars. Therefore, as suggested by Miller et al. [18] the level of γ-oryzanol and steryl ferulates, as a composition, in rice bran can be different by the effects of cultivar, such as growing location and grain maturity. In addition, Massarolo et al. [19] reported increase of γ-oryzanol content in rice bran of fermented with *Rhizopus oryzae*.

### 3.2. Method Validation for Determination of γ-Oryzanol

**Figure 2:**
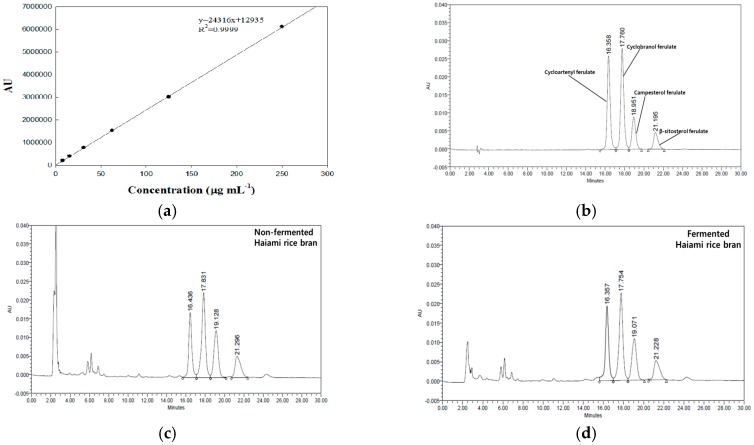
Linearity and specificity of γ-oryzanol. Calibration curve of γ-oryzanol standard solution (**a**); HPLC-PDA chromatograms of γ-oryzanol standard (**b**); non-fermented Haiami rice bran (**c**); and fermented Haiami rice bran (**d**).

The standard chromatograms of γ-oryzanol obtained using the HPLC-PDA methods are shown in Figure 2. The analyte was separated on a Sunfire C18 column, such as cycloartenyl ferulate, cyclobanol ferulate, campesterol ferulate and β-sitosterol ferulate peaks. In addition, interferences were not observed from the chromatograms (Figure 2b,c,d). The linearity of the method was evaluated over the range (7.8125–250 μg/mL) during validating (Figure 2a). The correlation coefficient (*R*^2^) showed 0.9999. The LOD and LOQ were 1.5816 and 4.7927 μg/mL, respectively. Similarly, Lu et al. [13] an used HPLC method to validate simultaneous quantification of five major triterpene alcohols and sterol ferulates in rice bran oils. These authors 0.13–0.20 μg/mL and 0.50–0.60 μg/mL of LOD and LOQ from isolated and purified γ-oryzanol, respectively.

The result for the recovery study of γ-oryzanol is summarized in Table 2. The precision of γ-oryzanol analysis was estimated by the relative standard deviation (RSD) for both intra-day and inter-day. Intra-day as well as inter-day precisions for γ-oryzanol of non-fermented Haiami rice bran were ranged within 0.44%–2.74% and these of fermented Haiami rice bran on both days were calculated within 0.12%–2.23%. The spiking levels chosen among recovery studies were 50, 60, 75 μg/mL in non-fermented and fermented Haiami rice brans. In non-fermented and fermented Haiami rice brans, the average recovery rates of γ-oryzanol were 107.11%–110.99% on intra-day and 107.83%–111.85% on inter-day, respectively.

### 3.3. Total Phenol Content of Non-Fermented and Fermented Rice Brans

Polyphenolic compounds including flavonoids are widely found in food products derived from plant sources and have been shown to possess significant antioxidant activities [20]. Total phenol contents of non-fermented and fermented rice brans varied from 31.32 to 156.08 mg GAE/g (Table 3). The highest content of total phenol was the fermented Haedam rice bran (No. 7, 156.08 ± 5.12 mg GAE/g). In addition, the fermented Heonpum rice bran (No. 6, 140.04 ± 1.00 mg GAE/g), the fermented Sunpum rice bran (No. 5, 139.68 ± 2.86 mg GAE/g) and the fermented Chindeul (No. 2, 137.41 ± 1.89 mg GAE/g) had higher in total phenol content than those of other cultivars. Meanwhile, the non-fermented Danmi rice bran was lowest level of total phenol contents (31.32 ± 0.32 mg GAE/g). Similar research was reported that the methanol extract of the fermented rice brans with *Rhizopus oligosporus* and *Monascus purpureus* showed highest total phenol content compared with the non-fermented rice brans [21]. 

### 3.4. Β-Glucan Content of Non-Fermented and Fermented Rice Brans

β-glucans are abundant in cell walls in cereal grains, mushrooms and yeasts. Generally, cereal grains contain mainly (1–3)(1–4)-β-glucans whereas mushrooms contain (1–3)(1–6)-β-glucans [22]. The widely reported nutritional benefit of β-glucan in foods is the control of the postprandial blood glucose and insulin rises [23]. β-glucan contents of non-fermented and fermented rice brans were measured a mixed-linkage β-glucan assay kit. Table 3 shows that the β-glucan contents in non-fermented and fermented rice brans ranged from 0.14% to 0.57%. Non-fermented Danmi rice bran (No. 12, 0.57% ± 0.01%) had the highest β-glucan content, followed by non-fermented Goami2 rice bran (No. 13, 0.44% ± 0.01%) and non-fermented Misomi rice bran (No. 15, 0.40% ± 0.03%). Fermented Chujum rice bran (No. 8, 0.14% ± 0.01%) was lower in β-glucan content than those of other cultivars. These results indicated that the β-glucan contents of fermented rice brans (except fermented Wolbaek and Anda rice bran) decreased by fermentation. Angelov et al. [24] reported after fermentation, that it was estimated that β-glucan content in the oat mash has not changed significantly with any of the latic acid bacteria and yeast. However, Kim et al. [25] showed that the β-glucan content was increased in black rice bran fermented by *L. edodes*. Meanwhile, Han et al. [26] reported that over-expression of β-glucanase enzyme decreased the level of β-glucan in grain.

### 3.5. Antioxidant Activity of Non-Fermented and Fermented Rice Brans

The stable radical DPPH is commonly used to evaluate the synthetic antioxidant as well as the antioxidant of natural phenolic compounds [27]. DPPH accepts an electron or hydrogen radical to become a stable diamagnetic molecule and reduced free radical form from the scavenger [28]. Table 4 shows the DPPH radical scavenging activity of non-fermented and fermented rice brans. DPPH radical scavenging activities ranged from 29.23% to 65.13% and from 33.53% to 71.30% in non-fermented and fermented rice brans, respectively. Most of the DPPH radical scavenging activity was higher in the fermented rice bran. The highest activity was in fermented Haedam rice bran (No. 7, 71.30% ± 0.78%) and fermented Haepum (No. 10, 70.43% ± 1.05%), followed by fermented Haepum rice bran (No. 10, 70.43% ± 1.05%) and fermented Misomi rice bran (No.15, 66.53% ± 0.72%). The non-fermented Danmi rice bran (No. 12, 29.23% ± 0.55%) was lower in DPPH radical scavenging activity than those of other cultivars. After bioconversion, DPPH radical scavenging activities were increased in most fermented rice brans (except fermented Gopum, Anda, Goami4 and Samkwang rice bran). Similarly, Bernaert et al. [29] reported an increase of the DPPH radical scavenging activity in leek upon fermentation. Rice bran is natural antioxidants that can be used as free radical scavengers and the strong antioxidant activity of rice bran extracts might be due to the presence of tocopherols and tocotrienols [30].

The ORAC assay directly measures the chain-breaking antioxidant capacity against peroxyl radicals, which is most relevant to the human body [31]. Table 4 shows the ORAC values in non-fermented and fermented rice brans. The ORAC values ranged from 220.93 to 1101.31 μM TE/g. The fermented Haedam rice bran had the highest ORAC value (No. 7, 1101.31 ± 41.11 μM TE/g), followed by fermented Misomi rice bran (No. 15, 943.68 ± 3.21 μM TE/g) and fermented Migwang rice bran (No. 18, 868.15 ± 17.84 μM TE/g). In addition, the lowest ORAC value was expressed from non-fermented Wolbaek rice bran (No. 9, 220.94 ± 4.88 μM TE/g) and non-fermented Goami4 rice bran (No. 16, 232.83 ± 2.23 μM TE/g), non-fermented Danmi rice bran (No. 12, 234.90 ± 4.78 μM TE/g). All of the ORAC values of rice bran samples were increased after bioconversion. Similarly, Escudero-López et al. [32] reported about the determination of ORAC value in the fermented orange juice. The ORAC value was increased from 6,044 to 8,229 or 7,889 μmol following by fermentation time. Cai et al. [33] also reported that the ORAC value of oats were increased through the fermentation by three filamentous fungi (*Aspergillus oryzae* var. *effuses*, *A. oryzae* and *A. niger*). Therefore, bioconversion has improved the antioxidant activity of rice bran when evaluated by the ORAC assay.

### 3.6. Relationship between Antioxidant Activities and Bioactive Compounds

**Figure 3:**
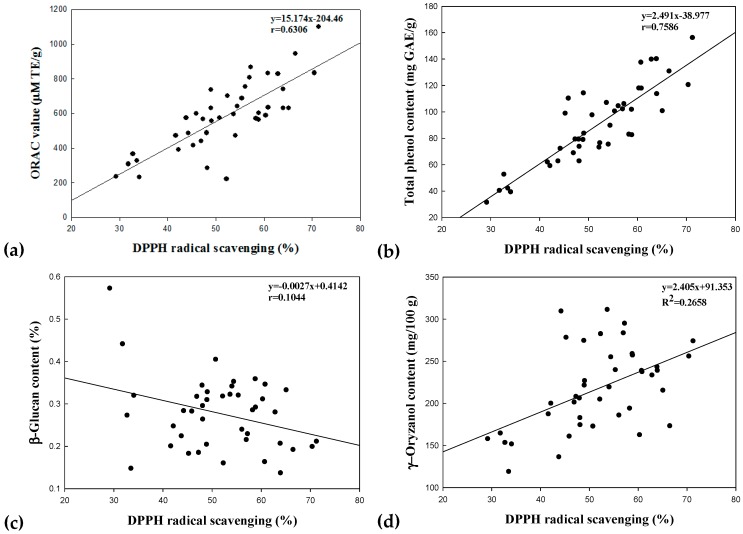

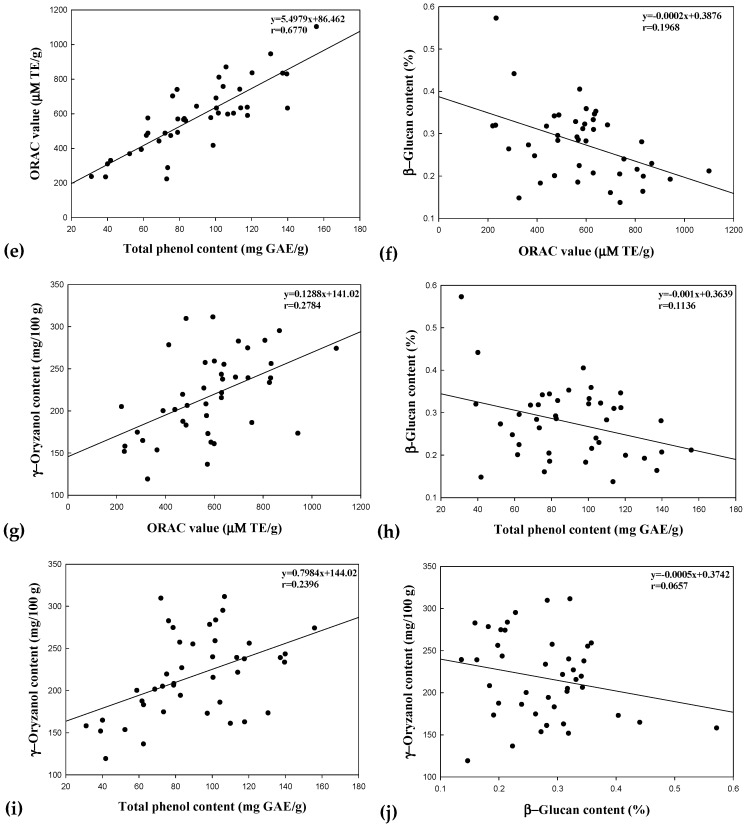
Linear correlation between the antioxidant activities and bioactive compounds. DPPH radical scavenging activity and the ORAC value (**a**); DPPH radical scavenging activity and the total phenolic content (**b**); DPPH radical scavenging activity and the β-glucan content (**c**); DPPH radical scavenging activity and the γ-oryzanol content (**d**); ORAC value and total phenolic content (**e**); ORAC value and the β-glucan content (**f**); ORAC value and the γ-oryzanol content (**g**); total phenolic content and the β-glucan content (**h**); total phenolic content and the γ-oryzanol content (**i**); and β-glucan content and the γ-oryzanol content (**j**). All of correlations among the response variables were significant as *p* < 0.05.

Correlation between antioxidant activities (DPPH radical scavenging activity and ORAC value) and bioactive compounds (γ-oryzanol, β-glucan and total phenol contents) of non-fermented and fermented rice brans was evaluated in this study. Figure 3 shows the correlation coefficients (*r*). 

The correlations among DPPH radical scavenging activities, ORAC values, total phenol contents, β-glucan contents and γ-oryzanol contents ranged between 0.0657 and 0.7586, with the highest correlation between DPPH radical scavenging activity and total phenol content (*r* = 0.7586, Figure 3b). Louaileche et al. [34] reported the scavenging capacity against the DPPH radical used for determining antioxidant potential that has been proven to exhibit a positive linear correlation (*r* = 0.77) with phenolic compound content. They stated that these compounds contributed to the antioxidant capacities of the different dated-varieties. Additionally, ORAC value, total phenol contents (*r* = 0.6770, Figure 3e), ORAC value and DPPH radical scavenging activity (*r* = 0.6306, Figure 3a) were highly correlated. Likewise, Thaipong et al. [35] reported a correlation between the ORAC value and DPPH radical scavenging activity of guava extracts (*r* = 0.68). The lowest correlation was found between the β-glucan content and γ-oryzanol content (*r* = 0.0049, Figure 3j). In addition, β-glucan content did not positively correlated with others (0.1044, 0.1136 and 0.1968 with DPPH radical scavenging activity, total phenol content and ORAC value). Similarly, the low correlations were also observed between the γ-oryzanol content and the analytic methods for antioxidant capacity (0.2396, 0.2658 and 0.2784 with total phenol content, DPPH radical scavenging and ORAC value). Bagchi et al. [36] mentioned low correlation between γ-oryzanol content and total phenol content (0.2058) in rice grain.

## 4. Conclusions

In conclusion, this study assessed and compared the bioactive compounds (γ-oryzanol, β-glucan and total phenol contents) and antioxidant activities of the 21 bioconversed rice bran cultivars. To measure the γ-oryzanol content of rice brans, the HPLC method was validated by linearity, specificity, accuracy, precision, limit of detection and limit of quantification. We suggest that the bioconversion process used in this study may amplify the antioxidant content in rice barn and be a potential way to produce the natural antioxidant agent from rice bran.

## Figures and Tables

**Table 1:** The characteristic of rice bran by cultivars.

Sample No.	Cultivar
1	O. *sativa* cv. Segyejinmi
2	O. *sativa* cv. Chindeul
3	O. *sativa* cv. Seolgaeng
4	O. *sativa* cv. Gopum
5	O. *sativa* cv. Sunpum
6	O. *sativa* cv. Heonpum
7	O. *sativa* cv. Haedam
8	O. *sativa* cv. Chujum
9	O. *sativa* cv. Wolbaek
10	O. *sativa* cv. Haepum
11	O. *sativa* cv. Anda
12	O. *sativa* cv. Danmi
13	O. *sativa* cv. Goami2
14	O. *sativa* cv. Dasan1
15	O. *sativa* cv. Misomi
16	O. *sativa* cv. Goami4
17	O. *sativa* cv. Ilpum
18	O. *sativa* cv. Migwang
19	O. *sativa* cv. Samkwang
20	O. *sativa* cv. Jungsaenggold
21	O. *sativa* cv. Haiami

**Table 2:** Accuracy and precision of γ-oryzanol analysis for *Oryza sativa* cv. Haiami rice bran.

Sample	Concentration (μg/g)	Intra-Day			Inter-Day		
		Mean ± SD (μg/g)	Recovery (%)	RSD (%)	Mean ± SD (μg/g)	Recovery (%)	RSD (%)
Non-fermented Haiami	50	53.56 ± 1.20	107.11	2.25	54.19 ± 0.33	108.37	0.60
	60	65.80 ± 0.29	109.67	0.44	64.96 ± 1.78	108.26	2.74
	75	81.61 ± 1.41	108.81	1.73	81.61 ± 1.27	108.81	1.55
Fermented Haiami	50	53.61 ± 0.81	107.21	1.51	53.92 ± 1.20	107.83	2.23
	60	65.84 ± 0.98	109.73	1.49	66.70 ± 0.08	111.16	0.12
	75	83.24 ± 1.32	110.99	1.58	83.89 ± 1.67	111.85	1.99

**Table 3:** β-glucan content and total phenolic content of 21 cultivars of rice bran by bioconversion.

Sample No.	TPC ^1^ (mg GAE ^2^/g)		β-Glucan Content (% of Dry wt)	
	NRB ^3^	FRB ^4^	NRB	FRB
1. Segyejinmi	98.80 ± 3.07 ^k^	76.37 ± 1.94 ^no^	0.18 ± 0.01 ^t^	0.16 ± 0.02 ^u^
2. Chindeul	117.89 ± 1.32 ^de^	137.41 ± 1.89 ^b^	0.31 ± 0.01 ^gh^	0.16 ± 0.01 ^u^
3. Seolgaeng	68.82 ± 0.56 ^p^	114.12 ± 2.55 ^ef^	0.32 ± 0.01 ^fg^	0.31 ± 0.01 ^gh^
4. Gopum	62.67 ± 1.21 ^q^	110.15 ± 4.74 ^fg^	0.29 ± 0.01 ^hi^	0.28 ± 0.00 ^ijk^
5. Sunpum	117.76 ± 1.48 ^de^	139.68 ± 2.86 ^b^	0.35 ± 0.00 ^de^	0.28 ± 0.04 ^ijk^
6. Heonpum	82.84 ± 1.30 ^m^	140.04 ± 1.00 ^b^	0.28 ± 0.01 ^ij^	0.21 ± 0.02 ^pqr^
7. Haedam	89.62 ± 1.61 ^l^	156.08 ± 5.12 ^a^	0.35 ± 0.01 ^d^	0.21 ± 0.01 ^opqr^
8. Chujum	82.44 ± 1.08 ^m^	113.58 ± 1.37 ^ef^	0.29 ± 0.01 ^hij^	0.14 ± 0.00 ^v^
9. Wolbaek	73.07 ± 1.83 ^op^	101.74 ± 1.52 ^ijk^	0.32 ± 0.01 ^fg^	0.36 ± 0.01 ^d^
10. Haepum	100.36 ± 3.48 ^jk^	120.43 ± 1.34 ^d^	0.33 ± 0.01 ^ef^	0.20 ± 0.01 ^qrst^
11. Anda	73.64 ± 1.99 ^o^	52.54 ± 1.42 ^r^	0.26 ± 0.02 ^kl^	0.27 ± 0.01 ^jk^
12. Danmi	31.32 ± 0.32 ^t^	104.38 ± 2.24 ^ijk^	0.57 ± 0.01 ^a^	0.24 ± 0.02 ^mn^
13. Goami2	40.28 ± 0.74 ^s^	61.83 ± 1.12 ^q^	0.44 ± 0.01 ^b^	0.20 ± 0.01 ^qrst^
14. Dasan1	59.01 ± 1.00 ^q^	79.23 ± 1.09 ^mn^	0.25 ± 0.02 ^lm^	0.18 ± 0.01 ^st^
15. Misomi	97.53 ± 1.37 ^k^	130.70 ± 4.44 ^c^	0.40 ± 0.03 ^c^	0.19 ± 0.01 ^rst^
16. Goami4	39.19 ± 1.09 ^s^	42.00 ± 0.72 ^s^	0.32 ± 0.02 ^fg^	0.15 ± 0.01 ^uv^
17. Ilpum	75.28 ± 1.38 ^no^	102.05 ± 1.87 ^ijk^	0.34 ± 0.01 ^de^	0.21 ± 0.02 ^opq^
18. Migwang	106.90 ± 2.63 ^gh^	105.97 ± 1.42 ^ghi^	0.32 ± 0.01 ^fg^	0.23 ± 0.01 ^mno^
19. Samkwang	79.12 ± 1.57 ^mn^	62.62 ± 1.67 ^q^	0.34 ± 0.01 ^de^	0.22 ± 0.02 ^nop^
20. Jungsaenggold	83.59 ± 1.86 ^m^	100.43 ± 1.55 ^jk^	0.33 ± 0.01 ^efg^	0.32 ± 0.01 ^fg^
21. Haiami	72.16 ± 2.66 ^op^	78.85 ± 2.36 ^mn^	0.28 ± 0.01 ^ij^	0.20 ± 0.02 ^qrs^

^1^ Total phenol content. ^2^ Data are expressed as mg of gallic acid equivalents (GAE). ^3^ Non-fermented rice bran. ^4^ Fermented rice bran. Means with the different letters are significantly different (*p* < 0.05) by Duncan’s multiple range test.

**Table 4:** Antioxidant activity of 21 cultivars of rice bran by bioconversion.

Sample No.	DPPH (%)		ORAC Value (μM TE ^1^/g)	
	NRB ^2^	FRB ^3^	NRB	FRB
1. Segyejinmi	45.28 ± 1.14 ^op^	52.35 ± 1.08 ^jk^	414.92 ± 13.92 ^mn^	700.34 ± 7.69 ^f^
2. Chindeul	60.34 ± 0.36 ^e^	60.75 ± 0.31 ^de^	587.79 ± 6.85 ^ijk^	832.25 ± 4.74 ^d^
3. Seolgaeng	46.96 ± 1.08 ^mno^	49.02 ± 0.43 ^lm^	440.01 ± 13.05 ^m^	631.52 ± 3.49 ^gh^
4. Gopum	48.12 ± 0.74 ^mn^	45.93 ± 0.41 ^nop^	485.65 ± 3.75 ^l^	600.62 ± 12.84 ^hi^
5. Sunpum	60.86 ± 0.62 ^de^	62.89 ± 0.88 ^cd^	635.30 ± 10.53 ^g^	827.50 ± 9.70 ^cd^
6. Heonpum	58.30 ± 1.82 ^efg^	63.93 ± 1.55 ^c^	569.64 ± 8.06 ^ijk^	630.18 ± 20.67 ^gh^
7. Haedam	54.45 ± 1.56 ^ij^	71.30 ± 0.78 ^a^	641.02 ± 23.39 ^g^	1101.31 ± 41.11 ^a^
8. Chujum	58.90 ± 1.39 ^ef^	63.98 ± 1.44 ^c^	564.15 ± 21.95 ^jk^	739.53 ± 5.98 ^e^
9. Wolbaek	52.21 ± 0.97 ^jk^	58.84 ± 2.67 ^ef^	220.94 ± 4.88 ^r^	601.62 ± 23.25 ^hi^
10. Haepum	65.13 ± 1.57 ^bc^	70.43 ± 1.05 ^a^	630.46 ± 21.14 ^gh^	834.13 ± 26.05 ^d^
11. Anda	48.15 ± 1.45 ^mn^	32.76 ± 0.50 ^s^	286.09 ± 5.09 ^q^	366.68 ± 5.01 ^o^
12. Danmi	29.23 ± 0.55 ^t^	56.12 ± 0.47 ^ghi^	234.90 ± 4.78 ^r^	755.12 ± 30.82 ^e^
13. Goami2	31.84 ± 0.97 ^s^	41.64 ± 1.61 ^r^	308.03 ± 7.13 ^pq^	472.61 ± 10.10 ^l^
14. Dasan1	42.17 ± 0.38 ^qr^	47.31 ± 1.87 ^mno^	391.50 ± 5.49 ^no^	567.35 ± 5.25 ^jk^
15. Misomi	50.78 ± 1.29 ^kl^	66.53 ± 0.72 ^b^	575.04 ± 3.43 ^ijk^	943.68 ± 3.21 ^b^
16. Goami4	34.13 ± 0.05 ^s^	33.53 ± 0.97 ^s^	232.83 ± 2.23 ^r^	328.13 ± 7.42 ^p^
17. Ilpum	54.09 ± 1.49 ^ij^	57.03 ± 0.64 ^fgh^	471.64 ± 2.23 ^l^	808.81 ± 16.67 ^d^
18. Migwang	53.72 ± 0.54 ^ij^	57.28 ± 1.83 ^fgh^	595.55 ± 12.59 ^ij^	868.15 ± 74.84 ^c^
19. Samkwang	48.02 ± 1.03 ^mn^	43.81 ± 1.37 ^pqr^	490.15 ± 8.29 ^l^	573.11 ± 17.85 ^ijk^
20. Jungsaenggold	49.09 ± 0.73 ^lm^	55.39 ± 0.27 ^hi^	558.46 ± 10.11 ^k^	688.35 ± 12.56 ^f^
21. Haiami	44.27 ± 1.38 ^pq^	48.93 ± 0.99 ^lm^	485.84 ± 10.17 ^l^	738.03 ± 8.92 ^e^

^1^ Data are expressed as μM Trolox equivalents (TE). ^2^ Non-fermented rice bran. ^3^ Fermented rice bran. Means with the different letters are significantly different (*p* < 0.05) by Duncan’s multiple range test.
